# A Tool to Assess Bias, Racism, and Allyship in the Healthcare Learning Environment

**DOI:** 10.7759/cureus.81193

**Published:** 2025-03-25

**Authors:** Simran Shamith, Carolyn Giordano, Beverley A Crawford, Angela Jones, Kaitlyn Bleiweiss, Elizabeth Kachur

**Affiliations:** 1 Medicine, Drexel University College of Medicine, Philadelphia, USA; 2 Medical Education, Drexel University College of Medicine, Philadelphia, USA; 3 Clinical Restorative Dentistry, University of Pennsylvania School of Dental Medicine, Philadelphia, USA; 4 Nursing, Wilkes University Passan School of Nursing, Wilkes-Barre, USA; 5 Assessment and Evaluation, Drexel University College of Medicine, Philadelphia, USA; 6 Medical Education, Medical Education Development, Global Consulting, New York City, USA

**Keywords:** bias and allyship, dental education, healthcare education, learning environment, medical education, nursing education

## Abstract

Introduction: While healthcare students receive robust clinical training, social issues like bias and allyship are often overlooked. This study developed a new survey tool to assess how healthcare students perceive bias, racism, and allyship in their learning environment.

Methods: Using a validated scale as a frame from the John Hopkins Learning Environment Survey (JHLES), researchers created a new assessment tool to measure perceptions of bias, racism, and allyship across three different healthcare institutions representing medicine, dentistry, and nursing.

Results: A total of 212 responses were collected. The data showed that non-minority students had greater agreement on questions regarding equitable opportunities to excel, feeling free to speak up in clinical settings against bias, and overall perceptions of the learning environment. Men felt more comfortable speaking up against racial bias or feeling that someone would speak out on their behalf and reported higher feelings of acceptance within their learning environment.

Conclusion: This study developed and implemented a new survey tool to assess bias and racism across three medical education environments, medicine, dentistry, and nursing, demonstrating its relevance across fields with differing curricula and cultures. While small differences related to institution, minority status, gender, and class year were noted, the tool’s wide applicability provides a baseline for institutions seeking to assess bias within their learning environments.

## Introduction

While healthcare students have curricula dedicated to acquiring practical medical knowledge, there is a less dedicated curriculum to teach social issues, such as bias and allyship. These social issues are often taught in one-off lectures, shorter electives, or as self-directed explorations. We know, however, that learning about and addressing social issues can influence patient care [1].

Various national and in-house student surveys report that bias exists in the learning environment and indicate that these hostile educational settings, or even a negative perception of it, can increase the chances of burnout [2]. Furthermore, studies find that if programs promoted an environment of acceptance and respect for racial and ethnically diverse communities, students felt better prepared to serve a diverse client population [3]. For these reasons, measuring bias and racism in the learning environment is essential. Over the years, various instruments have been developed to measure students’ perceptions of their overall learning environment [4]. These include the John Hopkins Learning Environment Survey (JHLES) [5], the Dundee Ready Education Environment Measurement (DREEM) [6,7], the Dental Student Learning Environment Survey (DSLES) [8], the Veterans Affairs Learners’ Perceptions Survey (VA LPS) [9], the Postgraduate Hospital Educational Environment Measure (PHEEM) [10], and the Surgical Theatre Educational Environment Measure (STEEM) [11]. While these tools have been used to measure a student’s overall learning environment, few have measured responses specific to bias and fewer have been created to be used across different healthcare fields.

The purpose of this study was to create a new survey tool based on a previously validated survey instrument to assess students' perceptions of their learning environment as it relates to experiences of bias and allyship. These concepts are often taught in the course of medical education, but the aim of this study was to understand how they might impact a healthcare student's learning environment. Once the tool was created, the next goal was to use the tool to understand the differences between three healthcare professions at institutions with different cultures.

## Materials and methods

Study design

Exemptions from the Institutional Review Boards at the three participating institutions (Drexel University College of Medicine, Philadelphia, University of Pennsylvania School of Dental Medicine, Philadelphia, Wilkes University Passan School of Nursing, Wilkes-Barre) were obtained (Protocols #2305009924, #854266, and #661) prior to beginning the multi-school study. Informed consent was obtained as per the protocols. 

The survey developed for this study was adapted from the Johns Hopkins Learning Environment Scale (JHLES) and presents 12 new questions as shown in Table 1 with a Likert scale of level of agreement (1: strongly disagree - 4: strongly agree). The process of survey development utilized a panel of experts in the field of medical education and in assessing bias in medicine. Over the span of six months, meetings were held once a month to discuss the survey items and to decide on the final wording of the items. The tool also included demographic questions such as gender, race, ethnicity, and training level. The goal of the survey was to measure perceptions of biases and allyship in the learning environment of trainees in the healthcare professions. After the initial question development by a committee of 10 content experts, the artificial intelligence tool ChatGPT was used to identify ambiguous or unclear wording. With those suggestions from ChatGPT, the instrument was further validated by a focus group of 14 medical students. This focus group utilized students to provide feedback on item wording and clarification. The final survey that was used can be seen in Table 1.

**Table 1:** Modified JHLES Bias and Allyship Survey Questions JHLES: John Hopkins Learning Environment Survey

Item Number	Question
Q1	My school teaches basic and clinical sciences in an unbiased way.
Q2	My school provides equitable opportunities to excel for students of all racial backgrounds.
Q3	There are people at my school who will serve as my allies if I experience racial bias.
Q4	I feel confident serving as an ally to colleagues when racial bias has occurred.
Q5	There are people at my school who stand up for others when racial bias occurs.
Q6	If I personally experience racial bias at my school, I would inform supervisors and school leaders.
Q7	I feel free to speak up when I witness racial bias in a classroom setting.
Q8	I feel free to speak up when I witness racial bias in a clinical setting.
Q9	The majority of the faculty model anti-racist behaviors when teaching.
Q10	The majority of the faculty model anti-racist behaviors when providing clinical care.
Q11	I feel accepted at my school regardless of my racial identity.
Q12	Please rate your overall perception of the learning environment at your institution.

Recruitment

Students from three institutions representing schools of medicine, dentistry, and nursing were asked via a recruitment email to complete the anonymous survey instrument via the online survey platform, Qualtrics [12]. Surveys were taken between September and October 2023 across all three institutions in this study. The survey was open to all class years at the dental, medical, and nursing school and was voluntary to complete. The sample size for each institution is based on the number of students enrolled at each school. 

Data analysis

IBM SPSS Statistics for Windows, Version 29 (Released 2023; IBM Corp., Armonk, New York, United States) was used to compute t-tests and analyses of variance (ANOVAs) with a post hoc Tukey test to examine differences between healthcare professions, minority status, gender, and class years for each survey item. Levene’s statistic was utilized appropriately for the t-test, and for all items, equal variances were assumed (p<.05) except for item 12. In the case of item 12, the test statistic corresponding to Levene’s statistic where equal variances were not assumed was used. As shared above, Pearson correlation coefficients were applied to evaluate the relationship among the individual questions, including the overall learning environment rating and to establish the reliability of the survey tool.

## Results

Table 2 shows correlations ranging from r = 0.237 to 0.698, to look at question-by-question relationships on the tool. Feasibility of administration was demonstrated by using the instrument across three health profession schools.

**Table 2:** Modified JHLES Anti-racism and Allyship Survey Questions **Correlation is significant at the 0.01 level (2-tailed). JHELS: Johns Hopkins Learning Environment Scale

Item	1	2	3	4	5	6	7	8	9	10	11
Q1	-	-	-	-	-	-	-	-	-	-	-
Q2	.454**	-	-	-	-	-	-	-	-	-	-
Q3	.438**	.525**	-	-	-	-	-	-	-	-	-
Q4	.392**	.338**	.538**	-	-	-	-	-	-	-	-
Q5	.302**	.513**	.605**	.442**	-	-	-	-	-	-	-
Q6	.296**	.341**	.568**	.539**	.401*	-	-	-	-	-	-
Q7	.284**	.351**	.455**	.451**	.431*	.540**	-	-	-	-	-
Q8	.237**	.365**	.414**	.404**	.385*	.515**	.623**	-	-	-	-
Q9	.495**	.544**	.506**	.424**	.410*	.383**	.356**	.379**	-	-	-
Q10	.457**	.523**	.442**	.431**	.444*	.375**	.436**	.410**	.698*	-	-
Q11	.475**	.539**	.616**	.443**	.570*	.507**	.510**	.535**	.553*	.600*	-
Q12	.492**	.559**	.486**	.360**	.494*	.329**	.347**	.393**	.528*	.560*	.605*

Health profession differences

An ANOVA and a post hoc Tukey test were performed to look at differences between the institutions on each item, as shown in Table 3. On all questions except Q3 and Q7, the nursing school had a significantly higher mean score than one or both other institutions indicating higher agreement and satisfaction with those items. Q3, asking if students feel they have allies at school, and Q7, asking about speaking up in a classroom setting, had no differences between the institutions. On Q1, asking if the school teaches basic sciences in an unbiased manner, Q2, asking if the school provides equitable opportunities for all racial backgrounds, Q9, asking if faculty model anti-racist behaviors when teaching, and Q10, asking if faculty model anti-racist behaviors when providing clinical care, the nursing school had a significantly higher agreement compared to both the medical and dental school. On Q4, asking if students feel confident to serve as an ally, Q6, asking if students would report instances of racial bias, and Q12, asking about overall perceptions of the learning environment, the nursing school had significantly greater agreement than the dental school. Q5 asks if there are people at the school who would stand up in instances of racial bias and both the medical school and the nursing school had greater agreement than the dental school. Q8 asks if students feel free to speak up in a clinical setting and Q11 asks about feeling accepted regardless of the student’s racial identity. On both questions, the nursing school had a significantly higher agreement than only the medical school.

**Table 3:** ANOVA Between Institutions on Each of the Modified JHLES Allyship Questions *p<0.05 is considered significant JHELS: Johns Hopkins Learning Environment Scale; MD: Medicine; BSN: Nursing; DMD: Dentistry

	Medicine			Nursing			Dentistry					
Item	n	x	SD	n	x	SD	n	x	SD	F	p	Post hoc Tukey
Q1	103	3.17	0.63	68	3.47	0.53	40	2.98	0.73	8.820	0.000*	MD < BSN > DMD
Q2	102	3.28	0.65	68	3.54	0.53	40	3.03	0.58	9.749	0.000*	MD < BSN > DMD
Q3	100	3.32	0.69	69	3.41	0.58	38	3.13	0.7	2.126	0.122	ns
Q4	102	3.41	0.63	69	3.61	0.52	40	3.23	0.62	5.46	0.005*	BSN > DMD
Q5	100	3.43	0.61	69	3.3	0.63	38	2.97	0.72	7.12	0.001*	MD > DMD BSN > DMD
Q6	98	3.08	0.88	69	3.38	0.64	39	2.85	0.09	5.71	0.004*	BSN > DMD
Q7	102	3.01	0.87	67	3.13	0.69	39	2.85	0.74	1.63	0.198	ns
Q8	97	2.67	0.99	69	3.22	0.66	40	2.83	0.75	8.58	0.000*	MD < BSN
Q9	102	3.26	0.74	69	3.54	0.53	39	3.13	0.61	5.74	0.004*	MD < BSN BSN > DMD
Q10	98	3.31	0.58	67	3.58	0.53	36	3.00	0.68	12.09	0.000*	MD < BSN MD > DMD BSN > DMD
Q11	100	3.31	0.69	69	3.57	0.56	38	3.26	0.64	4.10	0.018*	MD < BSN
Q12	102	3.06	0.77	67	3.24	0.65	40	2.75	0.90	5.17	0.006*	BSN > DMD

There were 212 responses to the survey and Table 3 shows a breakdown of mean scores from each institution for each question. Results varied by school, but similar trends emerged. Mean scores on every question averaged above 2.8 for each institution indicating a higher level of agreement overall. When results from the three institutions were aggregated, the highest overall mean was 3.4 for Q4: “I feel confident in acting as an ally to colleagues when racial bias has occurred.” The lowest overall mean score was 2.9 on Q8, which refers to feeling free to speak up against racial bias specifically in a clinical setting.

Minority group analysis

An independent sample t-test analyzed differences between those belonging to a minority group vs those who were not part of a minority group and identified as Caucasian. Overall, on all questions except Q7, which asked about speaking up against racial bias witnessed in a classroom setting, the non-minority group had a higher mean score, indicating higher satisfaction and comfort in speaking up. There were six questions that had significant differences between these groups as shown in Table 4. These questions asked about equitable opportunities to excel in school (Q2), people who stand up against racial bias in school (Q5), feeling free to speak up in clinical settings against bias (Q8), feeling that faculty model anti-racist behaviors while teaching (Q10), feeling accepted regardless of racial identity (Q11), and overall perceptions of the learning environment (Q12). Cohen’s D was computed for effect size for all significant items, and all were small. There were no statistically significant differences between students who identified as Hispanic versus non-Hispanic.

**Table 4:** Independent t-Test for Non-minority vs Minority Students on the Modified JHLES *p<0.05 is considered significant JHELS: Johns Hopkins Learning Environment Scale

	Non-Minority			Minority					
Item	n	x	SD	n	x	SD	t	p	D
Q1	118	3.27	0.65	92	3.17	0.64	1.905	0.279	-
Q2	118	3.39	0.64	91	3.22	0.59	4.075	0.049*	0.28
Q3	115	3.37	0.61	91	3.23	0.72	1.002	0.124	-
Q4	118	3.48	0.60	92	3.38	0.63	0.061	0.227	-
Q5	117	3.42	0.59	90	3.16	0.70	0.002	0.004*	0.4
Q6	113	3.19	0.81	92	3.08	0.87	0.040	0.350	-
Q7	116	3.01	0.76	91	3.03	0.85	3.171	0.829	-
Q8	115	2.99	0.84	90	2.74	0.91	4.010	0.047*	0.29
Q9	119	3.39	0.68	90	3.26	0.66	0.727	0.164	-
Q10	114	3.43	0.59	86	3.23	0.63	0.655	0.024*	0.33
Q11	116	3.49	0.58	91	3.25	0.71	0.511	0.008*	0.37
Q12	116	3.18	0.71	92	2.90	0.84	2.503	0.010*	0.36

Gender differences

As shown in Table 5, on every question, male students had a higher mean score, indicating higher agreement on each question. A t-test was conducted to compare mean scores on each question between male students and female students and Cohen’s D was computed on significant items. The effect size was small to moderate for each. On Q3-7, male students had a significantly higher mean score. These questions dealt with speaking up against racial bias or feeling that someone would speak out on your behalf. In addition, on Q9 and Q10, male students had a significantly higher mean score, and these questions focused on perceptions of faculty modeling anti-racist behaviors when teaching. Finally, male students had a significantly higher score on Q11, which asks about feeling accepted at your school regardless of your racial identity. Q1 and Q2 did not have significant differences between genders which ask about schools teaching basic science in an unbiased way and providing opportunities for all students to excel. Like the comparison between institutions, there was no significant difference on Q8 which asks about feeling free to speak up in a clinical setting.

**Table 5:** Independent t-Test for Female vs Male Students on the Modified JHLES *p<0.05 is considered significant JHELS: Johns Hopkins Learning Environment Scale

	Female			Male					
Item	n	x	SD	n	x	SD	t	p	D
Q1	151	3.23	0.62	57	3.26	0.70	-0.38	0.70	-
Q2	150	3.30	0.61	57	3.37	0.67	-0.70	0.48	-
Q3	147	3.22	0.66	57	3.56	0.60	-3.44	0.00*	0.54
Q4	152	3.39	0.62	56	3.57	0.57	-1.93	0.05*	0.30
Q5	148	3.24	0.64	57	3.51	0.63	-2.73	0.01*	0.43
Q6	146	3.07	0.84	57	3.35	0.79	-2.20	0.03*	0.34
Q7	148	2.95	0.76	57	3.21	0.88	-2.08	0.04*	0.32
Q8	146	2.83	0.85	57	3.04	0.94	-1.51	0.13	-
Q9	150	3.25	0.69	57	3.54	0.57	-2.84	0.00*	0.46
Q10	142	3.28	0.62	56	3.52	0.57	-2.46	0.01*	0.40
Q11	148	3.33	0.64	57	3.53	0.66	-1.94	0.05*	0.31
Q12	149	3.05	0.74	57	3.11	0.88	-0.48	0.63	-

Class year differences

Mean scores on each question were stratified by academic class year when scores from all three institutions were combined. The responses to the survey ranged between 35 and 62 students in each class year. An ANOVA with a post hoc Tukey test was conducted to compare mean scores across the class years. Only Q5 and Q12 had statistically significant differences with year 4 scoring significantly lower than the other three years. Q5 asks about the feeling that there are people at their school who would stand up for others in cases of racial bias and Q12 asks about the overall perception of the learning environment at their institution. Year 4 responders had the lowest average scores, while the scores of years 1-3 were comparable.

Correlation

Pearson correlations were run to observe associations between scores on each of the 12 questions. As shown in Table 2, correlation coefficients ranged from r =.237 to .698. The small to moderate correlations show a relatively reliable instrument. Q12 asks about a student’s overall perception of the institution, which was highly correlated with Q11, which asks about feelings of acceptance regardless of a student’s racial identity. The highest correlation was r=.698, p<.01, between Q9 and Q10, which ask if faculty demonstrate anti-racist behaviors when teaching versus when providing clinical care.

## Discussion

The purpose of this research was to develop and use a novel survey instrument to assess students’ perceptions of their learning environment as it relates to experiences of bias, racism, and allyship. School responses were compared in the areas of minority versus non-minority, male versus female, class years, and type of institution. When all responses were aggregated, the highest mean score was recorded for Q4: “I feel confident serving as an ally to colleagues when racial bias has occurred”. This indicates a high relevance of allyship overall, but further analysis of the mean score finds that the lowest score is speaking up in a clinical setting. This may indicate that as students come closer to graduating and in clinical scenarios, they are less inclined to be drawn into situations with people who may have control over their future. Looking at the institutional differences, the nursing school had significantly higher agreement than one or both other institutions on most questions. Faculty at the nursing school suggested that differences may be due to nursing students feeling more comfortable in the clinical environment due to beginning intensive clinical practice in their second year rather than their third year. Another consideration is that nursing students are less dependent than dental or medical students on letters of reference that may significantly impact their future careers. Nursing care is more often in-patient and lasts for an extended time, so nursing students may also learn to be strong advocates for their patients and themselves sooner than medical or dental students.

The ANOVA indicated similar levels of agreement to speaking up in a classroom setting for all three schools. This could indicate that the classroom setting appears less intimidating in the presence of peers, familiar faculty members, and the absence of patients, allowing students to feel comfortable speaking up. Furthermore, based on class years, years 1-3 had similar mean scores and year 4 had the lowest agreement on questions of speaking up and overall perception of the learning environment. In year 4, all students are in a clinical environment where there are patients, attending physicians, dental faculty, nurses, and other staff which can limit opportunities for speaking up. Williams and Sharif note that allyship behaviors tend to be conditional, and those who claim the title of ally do not always act when the situation to do so presents itself [13]. Dancis and Coleman categorize “transformative dissonant encounters” as meaningful personal experiences that can force an individual to confront their belief that racism is not a significant factor in society and their own experiences [14]. These experiences can raise awareness in future healthcare workers and acknowledging these experiences can create better allies. 

In comparing male allies to female allies, persons self-identifying as male scored significantly higher on questions about speaking up as an ally against racial bias. These results were different from the conclusions seen in previous research. Yuce et al. found in their research evaluating race and ethnic discrimination in US surgical residency programs that female residents were more likely to report discrimination than their male peers [15]. Hu et al., when similarly evaluating discrimination in surgical residency programs, found that gender discrimination and racial discrimination were reported by a higher percentage of women [16]. However, the actual act of speaking up may be different than the perceived comfort of speaking up that was assessed in this survey. There also may be confounders when considering the intersectionality of race and gender that was not assessed in this study due to sample size.

Similarly, non-minority students had scores indicating greater comfort in speaking up in situations of bias and feeling comfortable in their current learning environments across all three schools. In the overall population of healthcare students, bias and racism are documented as contributing to attrition, difficulty transitioning to professional practice, impaired therapeutic relationships, and self-doubt regarding academic and clinical competence [17-21].

This study developed a new survey tool to assess the prevalence and nature of bias and racism within diverse medical education environments. Testing the tool across three distinct disciplines, medicine, dentistry, and nursing, allowed the tool to be used in varying healthcare fields with differing curricula and cultures. While some differences emerged between schools, the tool demonstrated robust applicability across these environments without difficulty. The goal of this study was not to position one school above another but rather to validate the tool’s multidisciplinary utility. Importantly, this tool provides a baseline assessment that can guide institutions in identifying factors contributing to bias and in considering targeted changes. Its application across various contexts can generate informed interventions aimed at reducing bias and developing unbiased learning environments. Future studies may investigate a larger geographic area. It may be of utility to use this survey and changes in its scores to look at the impact of interventions at these institutions over time. 

Limitations

There are limitations to consider when evaluating the data of this study. First is the disparate number of responses between schools. The dental school had the lowest number of participants. The population of each school had significant differences in race and gender statistics. The survey results also express the views of a single educational institution relative to each profession. The three schools surveyed are located within approximately 100 miles of each other limiting the application of its results to farther geographic areas. Future studies including more institutions and more geographic diversity may provide more generalizable results.

## Conclusions

The value of this study was the creation of a novel survey instrument that concentrates on examining bias within educational settings across different health professions. This study demonstrated the survey’s utility and applicability across three diverse educational environments. The study found differences across all three schools involved, with more comfort in speaking up seen in the nursing school. In addition, this study found that male and non-minority students seemed more comfortable in their learning environments and in acting as allies. In healthcare training, bias contributes to self-doubt, burnout, and impaired patient relationships. Recommendations and strategies for dealing with bias include training on bystander intervention, championing advocacy, and engaging with affinity groups. Allyship can, however, provoke change, further cementing the importance of allyship and assessing bias in a healthcare learning environment.
